# Supplementary material for: Deletion of wfs1 Impairs Oligodendrocyte Precursor Cells Dorsal Distribution and Myelination Through the wfs1-hmgcs1 Axis in Zebrafish
Source: Biology (Basel). 2026 Aug 16;15(16):1402. doi: 10.3390/biology15161402 (PMC13510151; doi:10.3390/biology15161402)
Supplement: Supplementary file 1 [file biology-15-01402-s001.zip › File S1-Transcriptome sequencing data and differentially expressed genes (DEGs) in wfs1-- zebrafish at 5 dpf/2.KEGG/wfs1KOvsWT/GSEA/WT/MISMATCH_REPAIR/MISMATCH_REPAIR(DRE03430).html]

Details for gene set MISMATCH\_REPAIR(DRE03430)[GSEA]

|  || Dataset | deg.sample |
| Phenotype | sample.cls |
| Upregulated in class | WT |
| GeneSet | MISMATCH\_REPAIR(DRE03430) |
| Enrichment Score (ES) | -0.30981472 |
| Normalized Enrichment Score (NES) | -1.0485605 |
| Nominal p-value | 0.4352031 |
| FDR q-value | 0.578819 |
| FWER p-Value | 1.0 |
Table: GSEA Results Summary

  

Fig 1: Enrichment plot: MISMATCH\_REPAIR(DRE03430)      
 Profile of the Running ES Score & Positions of GeneSet Members on the Rank Ordered List

  

| PROBE | DESCRIPTION (from dataset) | GENE SYMBOL | GENE\_TITLE | RANK IN GENE LIST | RANK METRIC SCORE | RUNNING ES | CORE ENRICHMENT || 1 | 556995\_1 | lig1 |  |  | 956 | 2.157 | 0.0853 | Yes |
| 2 | 568323\_1 | mlh3 |  |  | 1446 | 1.693 | 0.1605 | Yes |
| 3 | 565400 | msh3 |  |  | 1596 | 1.597 | 0.2413 | Yes |
| 4 | 565400\_1 | msh3 |  |  | 2262 | 1.261 | 0.2878 | Yes |
| 5 | 556456\_1 | pold1 |  |  | 4702 | 0.718 | 0.2496 | No |
| 6 | 100037336 | rfc1 |  |  | 5563 | 0.631 | 0.2564 | No |
| 7 | 565276 | pms2 |  |  | 7515 | 0.453 | 0.2194 | No |
| 8 | 259256\_1 | rfc3 |  |  | 8868 | 0.377 | 0.1971 | No |
| 9 | 406778 | exo1 |  |  | 8984 | 0.369 | 0.2132 | No |
| 10 | 259256 | rfc3 |  |  | 9590 | 0.327 | 0.2117 | No |
| 11 | 393632 | mlh1 |  |  | 10059 | 0.300 | 0.2131 | No |
| 12 | 65226 | rpa2 |  |  | 10674 | 0.262 | 0.2078 | No |
| 13 | 406435 | rfc4 |  |  | 10835 | 0.253 | 0.2163 | No |
| 14 | 692256 | pold3 |  |  | 11051 | 0.241 | 0.2224 | No |
| 15 | 565276\_1 | pms2 |  |  | 12414 | 0.168 | 0.1887 | No |
| 16 | 406845 | msh2 |  |  | 12436 | 0.167 | 0.1970 | No |
| 17 | 556456 | pold1 |  |  | 13726 | 0.106 | 0.1622 | No |
| 18 | 796456 | pold2 |  |  | 13771 | 0.103 | 0.1663 | No |
| 19 | 260437 | msh6 |  |  | 13782 | 0.103 | 0.1715 | No |
| 20 | 556995 | lig1 |  |  | 14356 | 0.076 | 0.1576 | No |
| 21 | 393196 | rpa3 |  |  | 16744 | -0.024 | 0.0839 | No |
| 22 | 571834 | si:dkey-28b4.7 |  |  | 16805 | -0.027 | 0.0835 | No |
| 23 | 445385 | rfc5 |  |  | 16927 | -0.032 | 0.0814 | No |
| 24 | 327491 | rpa1 |  |  | 17594 | -0.058 | 0.0636 | No |
| 25 | 406778\_1 | exo1 |  |  | 17919 | -0.072 | 0.0572 | No |
| 26 | 30678 | pcna |  |  | 18983 | -0.121 | 0.0303 | No |
| 27 | 503748 | rfc2 |  |  | 22293 | -0.284 | -0.0584 | No |
| 28 | 550504 | ssbp1 |  |  | 22792 | -0.313 | -0.0572 | No |
| 29 | 556995\_2 | lig1 |  |  | 30840 | -1.799 | -0.2136 | No |
| 30 | 568323 | mlh3 |  |  | 31800 | -4.611 | 0.0028 | No |
Table: GSEA details [plain text format]

  

Fig 2: MISMATCH\_REPAIR(DRE03430)      
 Blue-Pink O' Gram in the Space of the Analyzed GeneSet

  

Fig 3: MISMATCH\_REPAIR(DRE03430): Random ES distribution      
 Gene set null distribution of ES for **MISMATCH\_REPAIR(DRE03430)**
